# The impact of trade facilitation on African SMEs’ performance

**DOI:** 10.1007/s11187-023-00756-4

**Published:** 2023-04-13

**Authors:** Andrew E. Hansen-Addy, Davide M. Parrilli, Ishmael Tingbani

**Affiliations:** 1 School of Business, Operations, and Strategy, Greenwich Business School, University of Greenwich, Old Naval College, Park Row, Greenwich, SE10 9LS UK; 2 Faculty of Management, Executive Business Centre, Bournemouth University, 89 Holdenhurst Road, Bournemouth, BH8 8EB UK; 3 Department of Accounting, Southampton Business School, University of Southampton, University Road, Building 2/ 6047Highfield Campus, Southampton, SO17 1BJ UK

**Keywords:** SMEs, Business Environment, Regulations, Institutional heterogeneity, Performance, Africa, L25, L26, O17, O24, O55

## Abstract

Whilst contemporary literature indicates that the business environment (BE) impacts almost all entrepreneurial activities, there are indications that the unique business and institutional setting in Africa (with its challenges and opportunities) and the nature of SMEs (their strengths and weaknesses), among other factors, lead to the context-specific impact of regulations on the performance of African SMEs. Using regressions and propensity score matching methods on a panel of 39,461 firm observations (27 African countries) from the World Bank Enterprise Surveys, we unearthed evidence to suggest that whilst enabling tax administration and business licensing regulations improve SMEs’ performance, trade facilitation impedes African SMEs’ performance. Furthermore, the institutional context of competition (from foreign firms) worsens trade facilitation’s negative impact on African SMEs’ performance. These findings suggest a fine-tuning of BE regulations in African countries. Trade facilitation, for example, must be carefully thought through and implemented in a way to benefit SMEs.

## Abstract (plain language summary)

Trade facilitation’s negative impact on African SMEs’ performance. This study contrasts trade facilitation’s impact on African SMEs’ performance with enabling tax administration and business registration regulations. The findings suggest that whilst enabling tax administration and business licensing regulations improve SMEs’ performance, trade facilitation impedes African SMEs’ performance. Moreover, the institutional context of competition (from foreign firms) exacerbates trade facilitation’s negative impact on African SMEs’ performance. This study’s main implications are: (1) trade facilitation and competition policies in Africa must be carefully thought through and implemented in such a way as to benefit SMEs. And (2) scholars may find useful the evidence that country-level proxies of regulations compliment consistently firm-level measures of regulations.

## Introduction 

Among the numerous developing countries, African countries have recently been of great interest to scholars and international organisations (Asongu & Odhiambo, 2019; Atiase et al., 2018; Dana et al., 2018; Kansheba, 2020) because these countries present a unique and challenging context for entrepreneurship. For example, poor access to finance and business development services (Brijlal, 2008; Fowowe, 2017; Mazanai & Fatoki, 2012), poor tax regimes (Adegboye et al., 2018; Adeniyi & Imade, 2018), corruption (d'Agostino et al., 2016), weak institutions (Alhassan & Kilishi, 2019), and inadequate infrastructure (Bond, 2016) are commonplace in African countries. In addition, SMEs contribute up to 70% of GDP in many countries and are often drivers of economic growth in developing countries (Ayyagari et al., 2007; Beck et al., 2005a, 2005b). They also represent more than 90% of businesses, significantly contribute to job creation, and have the highest proportion of sales and employment growth in African countries (Abor & Quartey, 2010; Ayyagari et al., 2014; World Bank, 2019a).

Consequently, there has been a keen interest in the literature and policy on the operations of SMEs in African countries (Atiase et al., 2018; Fatoki, 2014; Hunt et al., 2007; Kansheba, 2020; Sitharam & Hoque, 2016). Quite intriguing in the literature are the studies that have focused on the impact of the business environment (BE also referred to as “business climate,” “investment climate,” or “entrepreneurial ecosystem”) on SMEs’ performance. Factors such as the macroeconomic environment; infrastructure; security; political, social, and technological considerations; and the legal and regulatory framework generally determine the BE (Atiase et al., 2018; Belas et al., 2019; Dethier et al., 2011). The central theme of the literature on the BE is that it steers almost all entrepreneurial activities (Atiase et al., 2018; Audretsch et al., 2022; Braunerhjelm & Eklund, 2014; Chambers & Munemo, 2019; Kansheba, 2020; Klapper & Love, 2010; World Bank, 2004, 2020).

In this regard, some scholars have explored how Africa’s unique business and institutional setting impact entrepreneurship. For instance, Madzikanda et al. (2022) recently noted that weak entrepreneurial ecosystems diminished economic output and entrepreneurship in southern African countries, whereas Abubakar (2015) argued that the poor investment climate in Africa hindered entrepreneurship development. Similarly, Sheriff and Muffatto (2015) claimed that weak entrepreneurial ecosystems are responsible for poor entrepreneurship in Africa, and Munemo (2018) found that foreign direct investment (FDI) was less effective at enhancing entrepreneurship in African countries with less developed financial institutions and markets. Furthermore, contemporary studies indicate that BE regulations may have a context-specific impact on firm performance in Africa. For example, Kansheba (2020) noted mixed influences (positive and negative) of different BE elements (“eco-factors” such as governmental support and programmes) on African firms’ productivity. Moreover, although the literature confirms that conducive tax administration and business licensing and registration regulation^1^ improve African firms’ operations and performance (Adeniyi & Imade, 2018; Devas & Kelly, 2001; Kamasa et al., 2020), other regulations, such as trade facilitation (or liberalisation), raise queries (Hunt et al., 2007; Siddiqui, 2015; Terzİ, 2010). Whilst some firms may experience increased performance and export due to access to foreign consumers (Beverelli et al., 2015; Osakwe et al., 2018), other firms may limit their production and capital accumulation due to competition from imports when trade facilitation is encouraged (Bas & Ledezma, 2020).^2^ Some reasons for these mixed findings could be traced to firm size, industry, competition, and institutional setting (Hunt et al., 2007; Siddiqui, 2015; Terzİ, 2010). Trade facilitation thus carries some risks and may not always be beneficial to all firms. Nonetheless, two key insights are missing from the literature: (1) how trade facilitation impacts particularly African SMEs’ performance and (2) whether the institutional context of competition (from foreign firms) moderates trade facilitation’s impact on African SMEs’ performance.

This study, therefore, extends research on institutional heterogeneity and BE regulations’ impact on African SMEs’ performance. It aligns with the World Bank’s calls for such studies to inform policy directions (World Bank, 2020). This work takes a nuanced approach to regulations’ impacts on SMEs’ performance within the African context. We believe that not all standard enabling BE regulations, as established in previous studies, impact African SMEs in the same way. Africa’s unique business and institutional setting (with its challenges and opportunities) and African SMEs’ nature (their specific strengths and weaknesses) can imply regulations’ context-specific impact on SMEs’ performance. Thus, this study aims to contrast trade facilitation’s impact with other well-established enabling regulations’ impact on African SMEs’ performance.

To achieve this objective, we utilised a cross-country panel sample of 39,461 firms (covering 27 African countries) from the World Bank Enterprise Surveys (WBES). Using panel regressions and propensity score matching (PSM) methods, our findings suggest that not all enabling BE regulations enhance African SMEs’ performance. Indeed, our findings suggest that whilst enabling tax administration and business licensing regulations improve SMEs’ performance, trade facilitation impedes African SMEs’ performance. Furthermore, we note that the institutional context of competition (from foreign firms) exacerbates trade facilitation’s negative impact on African SMEs’ performance, which is argued based on institutional weaknesses and African SMEs’ constraints.^3^

This study makes three notable contributions to the literature on the institutional context and regulations in Africa. First, it provides first-time simultaneous evidence of the varied impact of enabling BE regulations on African SMEs’ performance. To our knowledge, no study has examined this evidence for SMEs or in the African context. Second, it provides new arguments and evidence demonstrating trade facilitation’s detrimental impact on SME performance in Africa. Third, it demonstrates that the institutional context of competition from foreign firms worsens trade facilitation’s detrimental impact on SMEs’ performance. In addition, by proxying regulations with objective country-level and subjective firm-level indicators, we provide a more thorough analysis of their impact and complementarity concerning firm performance.

The rest of this paper is structured as follows: Sect. 2 presents our theoretical arguments and hypotheses, whilst Sect. 3 clarifies the data and empirical methods we employed for our study. Section 4 then reveals the results and presents the discussion. Finally, Sect. 5 concludes the paper.

## Literature review and hypotheses

### The unique African business environment and institutional context for entrepreneurship

Policymakers and scholars have recently taken great interest in African countries because Africa presents a unique and challenging context for entrepreneurship (Atiase et al., 2018; Dana et al., 2018; Kansheba, 2020). For instance, despite the detrimental economic effects of the Russia-Ukraine war and the COVID-19 pandemic, the World Bank estimates that real GDP in sub-Saharan Africa would grow by 3.6% in 2023 and 3.9% in 2024, which are higher than the estimated 0.1% (2023) and 2.8% (2024) for Europe and Central Asia (World Bank, 2023). However, African countries face enormous challenges. For example, the African BE is considered one of the poorest in the world, with an average ease of doing business (EODB) score of 51.8, far below the global average of 63 (World Bank, 2020). Access to finance challenges (Fowowe, 2017), high youth unemployment (Chigunta, 2017), corruption (d'Agostino et al., 2016), crime (Wannenburg, 2005), weak institutions (Alhassan & Kilishi, 2019; Munemo, 2018), and inadequate infrastructure (Bond, 2016) are some pressing limitations in Africa which are also common in other developing countries (Agarwal & Mohtadi, 2004; Amirapu & Gechter, 2020; Beck, 2007; Dollar et al., 2005; Gnangnon, 2019; Mair & Marti, 2009; Nasrallah & El Khoury, 2022).

Moreover, entrepreneurship is promoted as a tool to alleviate some of the enormous challenges that developing African countries face (Naudé, 2010). Bruton et al. (2013) noted that promoting entrepreneurship and innovation in developing countries could alleviate poverty. Similarly, Kimhi (2009) observed that entrepreneurs’ rising income significantly reduced per capita household inequality in Ethiopia. Nafukho and Muyia (2010) argued that education and training in entrepreneurship are essential to reducing unemployment in Kenya.

In this regard, some scholars have shed light on the impact of Africa’s weak BE and institutional setting (voids)^4^ on entrepreneurship. Madzikanda et al. (2022) recently contended that unhealthy entrepreneurial ecosystems hindered economic output and entrepreneurship in southern African countries. Sheriff and Muffatto (2015) noted that African countries’ weak entrepreneurship environments (ecosystems) seem to be responsible for poor entrepreneurship in Africa. Using institutional theory, Atiase et al. (2018) observed that effective regulatory institutions (such as political governance and contract enforcement) are needed to support SMEs and entrepreneurship in Africa. Furthermore, Abubakar (2015) noted that the unfavourable investment climate and unavailability of entrepreneurship training impede entrepreneurship development in Africa. Nevertheless, African countries implementing economic reforms and macroeconomic management experience an improved investment climate that promotes greater entrepreneurship (Ahmed & Nwankwo, 2013; Atiase et al., 2018). Galperin and Melyoki (2018) thus argued that entrepreneurship policy implementation seems to be the missing link in improving the entrepreneurial ecosystem in Tanzania to support entrepreneurship.

African countries undoubtedly struggle with providing institutions to support their markets (Beck et al., 2008b). Inadequate access to formal business registration and support services, such as entrepreneurship capacity building (Atiase et al., 2018); weak economic institutions (Alhassan & Kilishi, 2019); poor access to essential finance and business development services, such as training and innovation (Brijlal, 2008; Fowowe, 2017; Mazanai & Fatoki, 2012); poor tax regimes, which include high taxes (Adegboye et al., 2018; Adeniyi & Imade, 2018); and poor and outdated labour regulations, such as minimum wage requirements, labour protection, and health and safety regulations (Kingdon & Knight, 2007; Nieuwenhuizen, 2019), are commonplace in Africa. When present, these institutional arrangements are often ineffective or obstructive (Xiaowei & Chi-Nien, 2013), and such constraints generally hinder SMEs’ operations and performance (Dethier et al., 2011; Weill, 2008). For instance, regulatory institutions responsible for licensing and permits for businesses in African countries are often ineffective, which leads to high numbers of unlicensed businesses, high start-up costs, and even business failures (Abor & Quartey, 2010; Devas & Kelly, 2001). In fact, the World Bank estimates that 21 days are needed to register a firm in sub-Saharan Africa, with an average of 7.4 procedures to complete, compared to 9.2 days and 4.9 procedures in OECD high-income countries (World Bank, 2020). This situation results in a permanent informality of firms, particularly SMEs, which prevents them from accessing several critical services (e.g., finance from banks, public subsidies for innovation, and training programmes) to expand their operations (Beck et al., 2008a, 2008b; Kansheba, 2020). Conversely, adequate access to business registration and other business support services is invaluable to SMEs’ survival and performance. For example, Devas and Kelly (2001) noted a marked improvement in local revenues and a reduction in compliance costs for firms in Kenya after numerous business licences were consolidated into a single business permit.

Complementarily, extant literature emphasises that African countries have poor tax regulation and administration systems that result in significant noncompliance with taxation (Adegboye et al., 2018). Businesses in African countries view tax regulation and administration as a burden on their businesses, stifling productivity to the extent that tax compliance requirements are considered a stumbling block for businesses in Nigeria and South Africa (Abrie & Doussy, 2006; Adegboye et al., 2018). According to Adeniyi and Imade (2018), there is a significant negative relationship between multiple tax burdens and the performance of businesses in Nigeria, due to poor tax administration. On the other hand, Kamasa et al. (2020) recently noted that sub-Saharan African firms’ productivity improved with better quality tax administration.

### Development of hypotheses

In this paper, these aforementioned contributions are recognised and used as a basis for the further refining of theory and policy implications in the context of developing African countries. Moreover, this paper takes a nuanced approach to regulations’ impact on SMEs’ performance, as not all standard regulations of an enabling BE are believed to improve SMEs’ performance in the African context. The unique African business and institutional environment, among other factors, may imply a mixed impact of different regulations on SMEs’ performance (Kansheba, 2020). One of these critical regulations is trade facilitation, which is generally considered a tool to spur economic growth in developing countries (Gnangnon, 2019; Osakwe et al., 2018). However, this regulation calls for a debate as contrary evidence suggests that trade facilitation carries some risks and is not always beneficial to all firms depending on firm size, industry, competition, and institutional setting (Hunt et al., 2007; Siddiqui, 2015; Terzİ, 2010). For example, whilst trade facilitation policies encourage export diversification in some developing countries (Beverelli et al., 2015; Osakwe et al., 2018), competition from imports harms the sales and capital accumulation of firms serving the domestic market in India (Bas & Ledezma, 2020). Furthermore, whilst trade facilitation in the form of reduction of input tariffs improved the productivity of firms in Brazil (Lisboa et al., 2010), small-scale farmers in some developing countries received limited gains from trade facilitation in the agricultural industry, with many farmers incurring increased costs (Wise, 2009).

Complementarily, studies in the African context, though not focused on SMEs, present mixed findings. For instance, Obuobi et al. (2022) argued that trade facilitation policies improved FDI inflows to African countries. Yameogo and Omojolaibi (2021) also argued that trade facilitation and institutional quality reduced poverty in sub-Saharan African countries in the long run, but noted that trade openness is detrimental to economic growth in the short term. Similarly, Mabugu and Mabugu (2014) found that trade facilitation improved technical factor productivity (TFP) and reduced poverty in South Africa only in the long term.

Nevertheless, African SMEs face numerous challenges that trade facilitation would exacerbate. For instance, by default, SMEs have a limited capacity to penetrate markets (Hussain 2000; Hashim and Wafa 2002). African SMEs find competing with large or foreign firms even more challenging when trade across borders is promoted (Sitharam & Hoque, 2016). Mutalemwa (2015) noted that competition from globalisation and weak institutional environments was damaging to the growth and development of African SMEs. Furthermore, Fatoki (2014) observed that external factors such as the rising cost of doing business, poor access to finance, competition, and the high cost of distribution led to the failure of new SMEs in South Africa. Ocloo et al. (2014) discovered that SMEs in Ghana were ill-prepared to embrace globalisation and competition. Hunt et al. (2007) noted that trade facilitation and increased competition had adverse effects on firms operating in the clothing sectors of Algeria, Morocco, and Tunisia. In fact, trade facilitation in these North African countries led to increased unemployment and hardships for workers and their households.

**Fig. 1:**
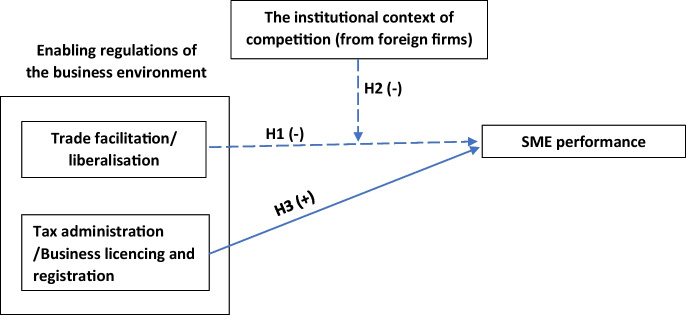
Conceptual framework

In essence, trade facilitation does not impact all firms’ performance in the same way. Whilst some firms may experience increased performance due to access to foreign consumers, others may limit their production and capital accumulation in response to import competition (Bas & Ledezma, 2020). We argue that even though trade facilitation offers firms some benefits, such as reduced production factor costs and increased access to foreign consumers, African SMEs are unable to utilise these benefits to improve their performance and that increased competition from foreign firms is detrimental to SMEs. Unlike large firms, SMEs do not have the capacity or leverage to compete against foreign firms, which adversely impacts their performance. This argument leads to our central hypothesis (Fig. 1):

- ***H1:*** Trade facilitation has a negative impact on African SMEs’ performance.

As posited hitherto, African SMEs are embedded in a context with specific features and limitations that impact their operations. Additionally, SMEs have limited resources and managerial capacity that limit the positive impact of trade participation. For instance, African SMEs face greater challenges in accessing finance than large firms (Quartey et al., 2017), have limited access to cutting-edge technology needed for upscale production (Maduku, 2021), limited knowledge-sharing networks to promote innovation (Mutalemwa, 2015), high production costs (Fatoki & Garwe, 2010), and often an unskilled labour force (Igwe et al., 2018), among other challenges. These impediments make competing with large firms, let alone foreign firms participating in their markets, challenging for African SMEs. Fatoki (2014) noted that the rising cost of doing business, competition and other factors led to the collapse of new South African SMEs. Similarly, Mutalemwa (2015) noted that African SMEs’ growth and development stalled due to globalisation and weak institutional environments. In essence, competition from foreign firms is an additional challenge African SMEs must deal with besides inherent setbacks that place them on a lower footing than large firms. We, therefore, argue that the institutional setting of competition (from foreign firms) in Africa worsens trade facilitation’s negative impact on African SMEs’ performance. This argument leads us to our second hypothesis:

- ***H2:*** The institutional context of competition (from foreign firms) exacerbates (or moderates negatively) trade facilitation’s negative impact on African SMEs’ performance.

Given the discussion hitherto, we find contrasting the impact of trade facilitation with the well-established impacts of other regulatory aspects, such as enabling tax administration and business licencing and registration regulations, on African SMEs’ performance appropriate. This step is essential to meeting this study’s objective. Thus, we include this third hypothesis.

- ***H3:*** Enabling tax administration, and business licencing and registration regulations have a positive impact on African SMEs’ performance.

## Methodology

### Data and sample

The sample for this study was constructed from firm-level data from the WBES and country-level data from the World Bank’s Doing Business project. The WBES, which began in 2002, is an extensive data repository that provides firm-level data for over 125,000 firms across 139 countries. The WBES data sets cover mainly firms in the manufacturing and service sectors and contain more than 100 BE indicators, such as firms’ access to finance, corruption, and performance measures (World Bank, 2019b). A sample based on available panel data sets on African countries was selected for this study. Twenty-eight panel data sets on Africa are available, and 27 were selected.^5^ The 27 panel data sets, which cover surveys conducted between 2003 and 2019, were appended to each other, yielding a rich unbalanced panel sample of 39,461 firm observations (see Table 1). The sample was limited to firms with up to 250 employees^6^ so that it aligned with other studies and the more general definition of SMEs, which is up to 250 employees (European Commission, 2020).

**Table 1:** Sample description

Country		Number of firms	Percentage	GDP per capita (USD)^a^	Ease of doing business score ^a^
1	Angola	593	1.79	2973.6	41.2
2	Benin	365	1.1	1219.4	51.7
3	Botswana	491	1.48	7961.3	66.2
4	Burkina Faso	443	1.33	774.8	51.3
5	Cameroon	675	2.03	1497.9	46
6	Cape Verde	197	0.59	3603.8	54
7	Chad	233	0.7	709.5	36.7
8	Cote d'Ivoire	739	2.23	2286.2	58.3
9	DRC	1388	4.18	545.2	35.2
10	Egypt	4689	14.12	3020.0	58.5
11	Ghana	1181	3.56	2202.1	60.4
12	Kenya	1991	6	1816.5	71
13	Liberia	220	0.66	621.9	43.5
14	Malawi	790	2.38	411.6	60.4
15	Mali	862	2.6	890.7	53.1
16	Morocco	2390	7.2	3204.1	71.7
17	Niger	302	0.91	554.6	52.3
18	Nigeria	7342	22.11	2229.9	53.4
19	Rwanda	643	1.94	801.7	75.4
20	Senegal	1677	5.05	1446.8	54.4
21	Sierra Leone	227	0.68	504.5	47.2
22	South Africa	1455	4.38	6001.4	66.7
23	Tanzania	1024	3.08	1122.1	54.3
24	Togo	245	0.74	675.5	55.3
25	Uganda	1098	3.31	776.8	58.4
26	Zambia	1048	3.16	1291.3	65.7
27	Zimbabwe	897	2.7	1464.0	50.5
	Total (*n*)	33,205	100		

The total sample size (*N*) is 39,461 observations

^a^World Bank values for 2019

The World Bank’s Doing Business project was launched in 2002 and measures business regulations’ influence on firms in over 190 countries and territories. The 10 main components of the overall EODB score include starting a business, dealing with construction permits, getting electricity, registering property, getting credit, protecting minority investors, paying taxes, trading across borders, enforcing contracts, and resolving insolvency. Two other areas (employing workers and contracting with the government) are not included in the EODB score (World Bank, 2020).

Each of the 10 components of the overall EODB score (excluding the ease of starting a business) has been calculated using at least two different methodologies since 2002. Based on availability and practicality, scores based on the 2006–2015 methodology for the ease-of-trading-across-borders, the 2004–2020 methodology for the ease-of-starting-a-business, and the 2006–2016 methodology for the ease-of-paying-taxe*s* were therefore selected as country-level measures of regulations and allocated to corresponding observations in the sample (see Table 2).

**Table 2:** Variables

Variable	Definition	Obs	Mean	Std. dev	Min	Max
Panel A: dependent variable (firm performance)						
Revenue	The log of the total annual sales of firm^a^	35,981	12.241	2.965	6.053	20.798
Panel B: objective independent variables (regulatory BE proxies)						
Ease of trading across borders	The regulatory BE proxied by the DB “ease-of-trading-across-borders” score	30,460	44.983	17.742	1.9	82.2
Ease of paying taxes	The regulatory BE proxied by the DB “ease-of-paying-taxes” score	34,069	53.270	13.827	14.9	78.6
Ease of starting a business	The regulatory BE proxied by the DB 'ease-of-starting-a-business’ score	38,457	66.498	17.299	17.4	93
Panel B: subjective independent variables (regulatory BE proxies)						
Customs and trade regulations	The regulatory BE proxied by how much of an obstacle customs and trade regulations are to a firm^b^	32,370	2.834	1.253	0	4
Tax administration	The regulatory BE proxied by how much of an obstacle tax administration is to a firm^b^	33,186	2.419	1.285	0	4
Business licensing & permits	The regulatory BE proxied by how much of an obstacle business licensing and permit regulations are to a firm^b^	32,790	2.807	1.191	0	4
Panel C: moderating variable						
The institutional context of competition (from foreign firms)	The institutional context of competition from foreign firms proxied by the KOF globalisation index	39,461	67.568	17.600	25.676	90.304
Panel D: control variables						
Retained earnings or internally gen. funds	Finance from retained earnings or internal funds^c^	37,764	3.244	1.046	1	4
Banks (public & private)	Finance from bank financial institutions, private and state-owned^c^	33,736	1.164	0.526	1	4
Access to finance constraints	Constraints (obstacles) in accessing external finance^d^	36,722	1.959	1.428	0	4
Size of firm	The size of a firm (measured by log of the number of permanent employees)	39,446	2.955	1.366	0	10.309
Age of firm	The log of the age of firm	38,411	2.480	0.872	0	5.352
Status of firm	Legal status of firm (1 = sole proprietorship; 2 = partnership; 3 = limited partnership; 4 = shareholding with traded shares; 5 = shareholding with non-traded shares; 6 = other)	37,721	3.034	1.005	1	6
Human capital of O/M	The human capital of the owner/manager (represented by the number of years of business-related experience)	38,169	14.758	10.431	0	72
Sector	The sector/industry of firm (1 = manufacturing; 2 = retail; and 3 = services)	31,911	1.821	0.884	1	3
Corruption	How much of an obstacle is corruption to a firm^d^	32,859	1.729	1.449	0	4
Informal firms	How much of an obstacle are practices of competitors in the informal sector^d^	36,806	1.774	1.391	0	4
Gross domestic product per capita	The log of the GDP per capita of the country where firm is located	39,461	7.353	0.756	5.543	8.769

^a^Annual sales is the converted USD equivalent using appropriate exchange rates from the International Financial Statistics (IFS) of the IMF

^b^0 = a very poor regulatory BE; and 4 = a very good regulatory BE

^c^1 = 0 to 25%; 2 = 26 to 50%; 3 = 51 to 75%; 4 = 76 to 100% of working capital

^d^0 = no obstacle; 1 = minor obstacle; 2 = moderate obstacle; 3 = major obstacle; 4 = very severe obstacle

### Variables

Table 2 describes the variables used for this study.

#### Dependent variables

Some of the standard measures of SME performance used in the literature are revenue, growth, profit, return on assets, return on investment, return on equity, and Tobin’s Q. We select revenue (that is annual sales of each firm) as the measure of SMEs’ performance following similar studies (Agostini et al., 2015; Fisman & Svensson, 2007; Otuo Serebour & Abraham, 2017; Xiang & Worthington, 2017) and available measures in the sample. All revenue and other monetary values in the sample were converted to equivalent USD values for each observation and year using corresponding exchange rates from the International Monetary Fund’s International Financial Statistics (IFS) (Bilgin et al., 2012).

#### Independent variables

Relevant regulations of the BE in this study are first proxied by three objective (country-level) Doing Business scores from the World Bank following similar studies (Bosire, 2019; Hossain et al., 2018; Munemo, 2012; Nketiah-Amponsah & Sarpong, 2020). These proxies are: (1) the ease-of-trading-across-borders score, (2) the ease-of-paying-taxes score, and (3) the ease-of-starting-a-business score. These three scores measure specific regulations’ impacts on businesses and correspond to trade facilitation, tax administration, and business licensing and permit regulations, respectively. These scores align with this study’s central hypothesis (see Appendix Table 10).

Furthermore, relevant regulations in this study are proxied by three subjective firm-level measures/variables (in the WBES sample) that cover the perceived impact of business regulations on SMEs following similar studies (Beck et al., 2005a, 2005b; Carlin et al., 2006; Commander & Svejnar, 2011). Firm-level measures are sometimes preferred over country-level measures because country-level measures cloud the heterogeneity usually present in each country or even in regions within a country (Dethier et al., 2011; Dollar et al., 2005). Country-level measures also fail to capture how different institutional deficiencies affect each unique firm because firms are not impacted in the same way (Straub, 2008). Three subjective firm-level measures of regulations were therefore used in this study (see Appendix Table 10). These measures are in response to the following question: How much of an obstacle do any of the following business regulations pose to a firm: (1) customs and trade regulations, (2) tax administration, and (3) business licensing and permits (these three measures correspond to trade facilitation, tax administration, and business licensing and permit regulation, respectively). A Likert scale range of responses sought are, ‘no obstacle’, ‘minor obstacle’, ‘moderate obstacle’, ‘major obstacle’, and ‘very severe obstacle’. This scale is reverted to reflect the quality of the regulatory BE; thus, ‘no obstacle’ = a very good BE (coded 4), ‘minor obstacle’ = a good BE (coded 3), ‘moderate obstacle’ = a moderate BE (coded 2), ‘major obstacle’ = a poor BE (coded 1), and ‘very severe obstacle’ = a very poor BE (coded 0).

#### Moderating variable

The institutional context of competition (from foreign firms) is proxied by the highly comprehensive KOF Globalisation^7^ Index (Gygli et al., 2019). This index, which the Swiss Economic Institute introduced in 2006, is computed from a wide range of development indicators from the World Bank, the International Monetary Fund, and other academic sources (Dreher, 2006). It measures the economic, social, and political dimensions of globalisation. For the economic dimension, indicators such as the export and import of goods and services, trade taxes, and FDI are included in the index, whilst for the social dimension, indicators such as international tourism and voice traffic, international patents, Internet and TV access, and press freedom are included. For the political dimension, the number of embassies in a country, the membership of international organisations, and international treaties are included (Gygli et al., 2019).

The KOF Globalisation Index is widely used in economics to measure globalisation, competition, institutional context or quality, and the development of countries (Bergh et al., 2014; Coulibaly et al., 2018; Doan, 2019; Potrafke, 2015; Shell & Zheng, 2015). We therefore find the KOF Globalisation Index an appropriate measure of the institutional context of competition from foreign firms in Africa.

#### Control variables

Various variables as controls were included in this study. First, we included variables that represented each firm’s funding source for working capital. These variables were working capital sourced from retained earnings and lending from banks. Due to obstructive financial systems in developing countries, retained earnings are the most popular funding source for SMEs (Bassetto et al., 2015; Zabri et al., 2015), and bank finance is the most accessible form of external funding available in developing countries (Beck, 2007; Quaye, 2014). Following Fowowe (2017), we also included a subjective measure of how accessible external finance is to firms.

Second, consistent with similar studies (Ebaid, 2009; Yazdanfar & Öhman, 2015) and the available variables in our WBES sample, we included variables that captured the firm’s character and the entrepreneur (or owner). These variables were the firm’s size, age, and legal status (for firm characteristics). Also included was the entrepreneur’s human capital (for entrepreneur characteristics). These firm characteristics are closely related to firm performance (Bilgin et al., 2012; Coad et al., 2013; Xiang & Worthington, 2015; Yuko et al., 2015).

Third, since the macroeconomic environment is considered another factor that impacts SMEs’ performance (Simerly & Li, 2000; Weill, 2008), we included the GDP per capita of the country where each firm is located at the time, *t*, as a country-level control (Fowowe, 2017; Ipinnaiye et al., 2017; Quartey et al., 2017). Also included was a measure of the competition registered SMEs face from informal firms. The informal sector is estimated to represent 40–50% of GDP in developing countries (Montenegro et al., 2010) and about 55–80% of GDP in African countries, which makes it a significant driver of economic growth in African countries (Abdelkader & Mansouri, 2022; Moyo & Sibindi, 2020).

Finally, this study includes a measure of corruption and its impact on SMEs’ operations. Corruption is an endemic problem that has a detrimental impact on the effectiveness of regulations and regulatory institutions, especially in developing countries (Hope, 2017; IMF, 2019; Olken & Pande, 2011).

### Econometric method

We undertook a few data cleaning operations to prime the sample for analysis, eliminating ambiguous entries in the data set, creating new panel IDs for the constructed sample, and recoding a few variables. Consistent with similar studies that aimed to determine firm performance (Dethier et al., 2011; Fowowe, 2017; Quartey et al., 2017), we used the following baseline model to explore firm performance as a BE function:

1 $${\mathrm{Performance}}_{it}={{\beta }_{0 }+ \beta }_{1 }{\mathrm{Business\;Environment }}_{it}{+ \beta }_{2 }{\mathrm{Controls }}_{it} +{v}_{it}$$

Here, the dependent variable, Performance, refers to the log of each firm’s annual revenue at a specific time, *t*. Business Environment refers to the set of country-level objective regulations (the ease-of-trading-across-borders score; the ease-of-paying-taxes score; and the ease-of-starting-a-business score) and firm-level subjective regulations (customs and trade regulations, tax administration, and business licencing and permits). Controls refers to a set of controls, including the firm’s sourcing from retained earnings and bank finance; access to finance; the firm’s size, age, and legal status; the human capital of the firm’s owner or manager; competition from informal firms; corruption; and the GDP per capita of the country where the firm operates. *V* refers to unobserved idiosyncratic errors. We first used the panel regression estimator for our initial econometric analysis following similar studies (Dethier et al., 2011; Dollar et al., 2005; Fowowe, 2017; Quartey et al., 2017). This method is suitable given our unbalanced panel data set’s nature.

Possible concerns with similar studies using the panel regression estimator include endogeneity, where the unobserved (time-invariant) error term (or omitted variable) is correlated with the regressors, which confounds the estimations (Cavaco et al., 2016; Ghosh, 2017; Wooldridge, 2016). Another possible concern is self-selection bias in the data collection process. To counter these problems, we utilised PSM methods to test for treatment effects of enabling regulations on SMEs’ performance. PSM methods are more effective in establishing causal relationships by disentangling the influence of the treatment (in this study, enabling regulations) from other covariates that may well influence SMEs’ performance (Phillipson et al., 2019). These methods also reduce selection bias, which may have occurred in the data collection process (Cepeda et al., 2003).

As treatment variables that correspond to the explanatory (independent) variables are needed to perform PSM analyses, we constructed three treatment variables from the distribution of the three objective (country-level) Doing Business scores (see Table 3). An obstructive regulatory BE (coded 0) referred to scores up to the 50^th^ percentile in each distribution, whereas an enabling regulatory BE (coded 1) referred to scores above the 50^th^ percentile in each distribution. Furthermore, we constructed additional treatment variables from the responses of the three subjective firm-level regulatory BE variables as follows: an obstructive regulatory BE (coded 0) referred to responses from firms that considered a specific regulation a ‘major obstacle’ or ‘severe obstacle’ to their operations, whilst an enabling regulatory BE (coded 1) referred to responses from firms that considered a specific regulation a ‘no obstacle’ or a ‘minor obstacle’ to their operations.

**Table 3:** Propensity score matching—construction of treatment variables

	Control (obstructive BE)	Treated (enabling BE)
Objective country level treatments (× 3)^a^	SMEs in locations with Doing Business scores up to the 50th percentile	SMEs in locations with Doing Business scores above the 50th percentile
Subjective firm level treatments (× 3)^a^	SMEs indicating regulations were a “major obstacle” or “severe obstacle” to their operations	SMEs indicating regulations were a “minor obstacle” or “no obstacle” to their operations

^a^These 3 treatment variables correspond to trade facilitation, tax administration, and business licensing and registration

We compared firms operating in obstructive regulatory BEs with firms operating in enabling BEs, matching firms by their sourcing from retained earnings and bank finance, access to finance, size, age, and legal status; the human capital of their owner or manager; the level of corruption where the firm operates, competition from informal firms, and the year of the survey. To ensure that a firm was not matched to itself in the panel data set, we ran our PSM models using *n*. We also included the year of survey in the matching criteria to ensure that matched firms were surveyed at about the same time to avoid, for instance, a firm that was surveyed in 2005 being matched to one that was surveyed in 2018.

The PSM process requires compressing the matching criteria (or covariates) into a single propensity score, calculated as the probability of treatment on the covariates. After propensity scores are obtained, individual firms with similar propensity scores can be compared (matched) across the control group (obstructive regulatory BE) and the treated group (enabling regulatory BE). Because propensity scores are estimated with a logit (or probit) model, our logit regression was formulated as follows.

2 $${\mathrm{Propensity\;score}=\mathrm{Pr}(T}_{i}=1)={{\beta }_{0 }+ \beta }_{1 }{Z}_{i} + {v}_{i}$$

Here, *T* is the binary treatment variable representing whether a firm is located in an obstructive (= 0) or enabling (= 1) regulatory BE, *i* refers to each firm in the sample, *Z* refers to the matching criteria or covariates used in this study, and *v* refers to the unobserved error.

The propensity scores, once computed, formed the basis for matching firms across the control and treated groups. We utilised these matching approaches to ensure consistency (Wooldridge, 2010), the nearest neighbour matching (or Mahalanobis distance matching) that Abadie and Imbens (2006) proposed, inverse probability weighting, and regression adjustment. After matching, a balancing test (which ascertains if there are no significant differences between covariate means across both control and treated groups) is also required (Dehejia & Wahba, 2002). Once the balancing test is successful, the average treatment effect on treated (ATET), which is the mean effect of firms that are treated (or those that are located in enabling regulatory BEs), can be computed (Wooldridge, 2010).

## Results and discussion

### Descriptive statistics

We noted interesting descriptive information on the nature of firms found in the sample. For instance, per the WBES’s classification of firms, 5.06% of the observations are micro firms (1–4 employees), 52.64% are small firms (5–19 employees), 28.26% are medium firms (20–99 employees), and 14.04% are large firms (100–250 employees). Moreover, 53.55% of firms are limited partnerships, whilst only 19.84% are ordinary partnerships and 5.16% are sole proprietorships. These statistics indicate an improvement in the general size and legal structure of registered firms in Africa (Abor & Quartey, 2010; Ayyagari et al., 2014; World Bank, 2019a). Also noteworthy is that most firms are in the manufacturing sector (49.66%), which covers industries such as textiles, garment making, plastics and rubber, fabricated metal products, non-metallic products, and chemicals. Firms engaged in services (31.75%) cover industries such as machinery and equipment, automobiles, and electronics, whereas those engaged in retail (18.6%) cover industries such as clothing, electronics, food, and household items. These statistics also indicate a notable shift from primary production to industrialisation in Africa (Abor & Quartey, 2010; Ayyagari et al., 2014).

### Empirical results

Table 4 presents the bivariate correlation matrix for all variables. A correlation of 0.90 and above is considered problematic; however, no significant correlations between these variables were observed. Table 6 presents the regressions underlying PSM analyses. The balancing tests on whether there are no significant differences between covariate means across both control and treatment groups were satisfied in almost all matching estimations, with differences in covariate weighted means negligible and variance ratios near 1.

**Table 4:** Correlations

	1	2	3	4	5	6	7	8	9	10	11	12	13	14	15	16	17	18	19
1	1																		
2	− 0.0603	1																	
3	0.3155	− 0.037	1																
4	0.181	0.4164	0.4441	1															
5	− 0.0696	− 0.0072	0.0827	0.0876	1														
6	0.0755	0.0203	0.1907	0.082	0.3475	1													
7	0.0879	0.0646	0.1093	0.1113	0.3358	0.3538	1												
8	− 0.0576	− 0.0897	− 0.0312	− 0.1167	0.0789	0.0682	0.054	1											
9	0.1446	0.0901	0.0827	0.0286	− 0.0985	− 0.0252	− 0.0046	− 0.4291	1										
10	− 0.1498	− 0.1207	− 0.1151	− 0.1203	− 0.1078	− 0.2337	− 0.2309	0.0218	− 0.0198	1									
11	0.4861	0.0375	0.1302	0.0305	− 0.1335	− 0.0143	0.0212	− 0.1111	0.1854	− 0.1688	1								
12	0.1471	0.1012	0.0454	0.0997	− 0.0759	− 0.0489	0.0333	− 0.0801	0.0989	− 0.0279	0.3512	1							
13	− 0.1082	0.0287	0.0161	0.0154	− 0.024	− 0.0074	− 0.0463	0.0608	0.0014	0.0626	− 0.0681	0.0021	1						
14	0.1344	0.1327	0.0156	0.0378	− 0.0847	− 0.0501	0.0014	− 0.0593	0.0914	0.0117	0.2265	0.5706	0.0408	1					
15	− 0.099	0.0072	− 0.1385	− 0.044	− 0.3148	− 0.3495	− 0.321	− 0.0711	0.0385	0.2077	0.0219	0.0544	0.0343	0.0743	1				
16	− 0.0488	− 0.0474	− 0.1371	− 0.1476	− 0.2371	− 0.2468	− 0.252	− 0.0202	0.0324	0.2468	− 0.04	0.0432	0.0513	0.0788	0.2765	1			
17	− 0.0225	0.0289	0.2178	0.3316	0.1831	0.1484	0.145	− 0.003	− 0.0592	− 0.0912	− 0.0126	0.078	− 0.0036	0.0023	− 0.1462	− 0.0603	1		
18	0.0069	0.4375	0.2411	0.5183	0.1295	0.166	0.0749	− 0.0731	− 0.0386	− 0.1401	0.003	0.0145	− 0.0208	− 0.0258	− 0.0192	− 0.1726	− 0.0144	1	
19	− 0.168	0.061	− 0.1153	0.4919	0.07	− 0.0897	0.0236	− 0.0812	− 0.0924	0.1069	− 0.1463	0.03	0.0268	− 0.0466	0.0675	− 0.0778	0.1876	0.213	1

1, revenue; 2, ease of trading across borders; 3, ease of paying taxes; 4, ease of starting a business; 5, customs and trade regulations; 6, tax administration; 7, business licensing and permits; 8, retained earnings; 9, bank finance; 10, access to finance; 11, size of firm; 12, age of firm; 13, status of firm

14, human capital of O/M; 15, corruption; 16, informal firms; 17, country; 18, GDP per capita; 19, KOFGI

**Table 5:** Regression results

Revenue	Model 1	Model 2	Model 3
Ease of trading across borders		− 0.040***	0.041***
		(0.002)	(0.005)
Institutional context of competition (from foreign firms) x Ease of trading across borders			− 0.001***
			(0.000)
Ease of paying taxes		0.019***	0.026***
		(0.002)	(0.002)
Ease of starting a business		0.076***	0.070***
		(0.002)	(0.002)
Customs and trade regulations		− 0.119***	− 0.138***
		(0.016)	(0.016)
Tax administration		0.064***	0.071***
		(0.016)	(0.016)
Business licensing and permits		0.118***	0.102***
		(0.016)	(0.016)
Retained earnings and internally gen. funds	0.031*	0.049***	0.052***
	(0.018)	(0.019)	(0.018)
Banks (public and private)	0.358***	0.241***	0.224***
	(0.036)	(0.041)	(0.041)
Access to finance	− 0.074***	− 0.037***	− 0.047***
	(0.012)	(0.013)	(0.013)
Size of firm	1.189***	1.100***	1.094***
	(0.017)	(0.018)	(0.018)
Age of firm	− 0.156***	− 0.194***	− 0.172***
	(0.025)	(0.027)	(0.027)
Status of firm	− 0.193***	− 0.276***	− 0.242***
	(0.018)	(0.020)	(0.020)
Human capital of O/M	0.180***	0.259***	0.246***
	(0.026)	(0.027)	(0.027)
Corruption	− 0.168***	− 0.165***	− 0.162***
	(0.012)	(0.014)	(0.013)
Informal firms	− 0.005	0.050***	0.047***
	(0.013)	(0.014)	(0.014)
Country	− 0.024***	− 0.063***	− 0.086***
	(0.003)	(0.003)	(0.003)
GDP per capita	0.114***	− 0.393***	− 0.459***
	(0.025)	(0.030)	(0.030)
Institutional context of competition (from foreign firms)	− 0.008***	− 0.032***	0.030***
	(0.001)	(0.001)	(0.004)
Constant	9.287***	11.335***	8.481***
	(0.230)	(0.251)	(0.303)
Wald’s Chi-square test	6816.11***	10,585.36***	10,918.02***
Observations	24,192	19,534	19,534
Number of firms	21,629	18,230	18,230

Standard errors in parentheses

^***^p < 0.01

^**^p < 0.05

^*^p < 0.1

Model is random effects

This study’s central hypothesis (H1) predicted that trade facilitation has a negative impact on African SMEs’ performance. The panel regression results (see Model 2 of Table 5; ease of trading across borders, β =  − 0.040, *p* < 0.01; customs and trade regulations, β =  − 0.119, *p* < 0.01) provide adequate support for this prediction, which means that, holding all other factors fixed, a one-unit increase in trade facilitation (proxied by the ease-of-trading-across-borders objective country-level measure) results in a 4% decrease in SMEs’ revenue. In addition, holding all other factors fixed, a one-unit increase in trade facilitation (proxied by subjective firm-level customs and trade regulations) results in an 11.9% decrease in revenue for SMEs. The ATET results of all PSM methods are statistically identical (see Tables 7–9). These statistics confirm the acceptance of H1.

**Table 6:** Probability of firm being located in an enabling regulatory BE

	Objective regulatory BE			Subjective regulatory BE		
	Ease of trading across borders	Ease of paying taxes	Ease of starting a business	Customs and trade regulations	Tax administration	Business licensing and permits
Retained earnings and internally gen. funds	0.020	0.607***	− 0.032	0.052**	0.159***	0.130***
	(0.022)	(0.061)	(0.021)	(0.020)	(0.018)	(0.020)
Banks (public and private)	0.466***	2.120***	0.199***	− 0.138***	0.142***	0.101**
	(0.051)	(0.149)	(0.043)	(0.038)	(0.036)	(0.040)
Access to finance	− 0.232***	− 0.164***	− 0.075***	− 0.103***	− 0.239***	− 0.283***
	(0.019)	(0.041)	(0.015)	(0.014)	(0.012)	(0.016)
Size of firm	− 0.106***	1.260***	0.062***	− 0.282***	− 0.037**	− 0.012
	(0.022)	(0.066)	(0.020)	(0.019)	(0.017)	(0.019)
Age of firm	0.110***	− 0.213**	0.306***	− 0.039	− 0.109***	0.132***
	(0.033)	(0.088)	(0.032)	(0.028)	(0.024)	(0.028)
Status of firm	0.223***	0.537***	− 0.066***	− 0.041**	0.032*	− 0.034*
	(0.026)	(0.071)	(0.022)	(0.020)	(0.018)	(0.021)
Human capital of O/M	0.552***	0.331***	0.164***	− 0.038	0.045*	0.061**
	(0.038)	(0.090)	(0.031)	(0.029)	(0.026)	(0.029)
Corruption	− 0.077***	− 0.322***	− 0.013	− 0.400***	− 0.453***	− 0.410***
	(0.016)	(0.041)	(0.015)	(0.014)	(0.012)	(0.017)
Informal firms	0.125***	0.033	− 0.059***	− 0.270***	− 0.193***	− 0.247***
	(0.017)	(0.044)	(0.015)	(0.014)	(0.013)	(0.016)
Country	0.012***	0.230***	0.109***	0.060***	0.047***	0.032***
	(0.003)	(0.011)	(0.005)	(0.003)	(0.003)	(0.003)
GDP per capita	1.558***	0.314***	2.509***	0.415***	0.348***	0.043
	(0.078)	(0.088)	(0.088)	(0.025)	(0.023)	(0.026)
Constant	− 14.569***	− 17.124***	− 21.129***	0.086	− 1.394***	1.613***
	(0.712)	(0.859)	(0.754)	(0.242)	(0.221)	(0.255)
Observations	22,400	23,903	26,362	21,747	20,488	20,904
Number of firms	20,541	21,446	23,281	19,684	18,577	18,970

Standard errors in parentheses

^***^p < 0.01

^**^p < 0.05

^*^p < 0.1

**Table 7:** ATET results of nearest neighbour matching

	Objective regulatory BE			Subjective regulatory BE		
	Ease of trade across borders	Ease of paying taxes	Ease of starting a business	Customs and trade regulations	Tax administration	Business licensing and permits
Nearest neighbour (3)	− 1.612***	1.198***	1.705***	− 0.250***	0.267***	0.242***
	(0.089)	(0.053)	(0.051)	(0.068)	(0.051)	(0.068)
Observations						
Total raw	18,685	19,454	21,170	17,728	16,586	16,946
Total matched	11,978	14,400	18,912	29,008	22,046	26,874
Treated matched	5989	7200	9456	14,504	11,023	13,437
Control matched	5989	7200	9456	14,504	11,023	13,437

Standard errors in parentheses

^***^p < 0.01

^**^p < 0.05

^*^p < 0.1

ATET is average treatment effect on the treated

The following covariates are included in all models: retained earnings, bank finance, access to finance, size of firm, age of firm, status of firm, human capital of O/M, corruption, informal firms, GDP per capita of country, and year of survey

**Table 8:** ATET results of inverse probability weighting

	Objective regulatory BE			Subjective regulatory BE		
	Ease of trade across borders	Ease of paying taxes	Ease of starting a business	Customs and trade regulations	Tax administration	Business licensing and permits
ATET	− 0.402***	1.241***	1.312***	− 0.313***	0.261***	0.280***
	(0.068)	(0.049)	(0.096)	(0.086)	(0.057)	(0.069)
POM^a^ (enabling reg BE)	12.544***	11.665***	11.297***	12.059***	11.752***	11.743***
	(0.055)	(0.027)	(0.092)	(0.084)	(0.052)	(0.066)
Observations						
Total raw	18,685	19,454	21,170	17,728	16,586	16,946
Total weighted	18,685	19,454	21,170	17,728	16,586	16,946
Treated weighted	9763.9	9701.1	10,192.6	8566.6	8227.2	8519.0
Control weighted	8921.1	9752.9	10,977.4	9161.4	8358.8	8427.0

Standard errors in parentheses

^***^p < 0.01

^**^p < 0.05

^*^p < 0.1

ATET is average treatment effect on the treated

The following covariates are included in all models: retained earnings, bank finance, access to finance, size of firm, age of firm, status of firm, human capital of O/M, corruption, informal firms, GDP per capita of country, and year of survey

**Fig. 2:**
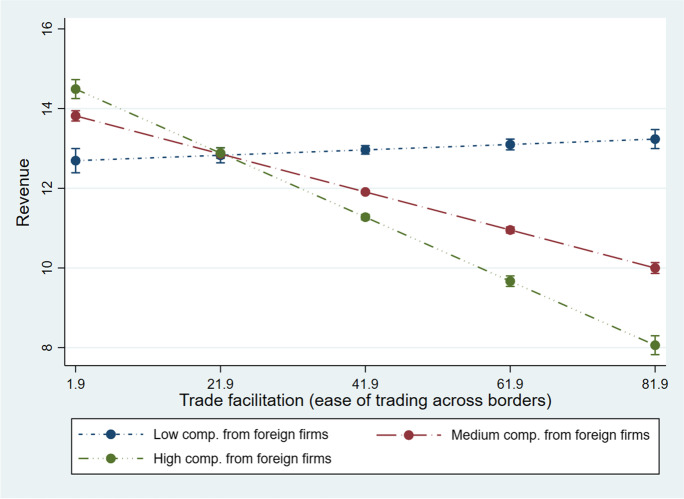
SMEs' performance: the interaction effects of the institutional context of competition and trade facilitation

The second hypothesis (H2) predicted that the institutional context of competition (from foreign firms) exacerbates (or moderates negatively) trade facilitation’s negative impact on African SMEs’ performance. Model 3 of Table 5 demonstrates that the institutional competition context’s moderating effect on the relationship between trade facilitation and African SMEs’ performance is negative and significant (β =  − 0.001, *p* < 0.01). Figure 2 shows the interactions between the institutional context of competition and trade facilitation on SMEs’ performance, affirming that revenue for SMEs in institutional contexts with low competition marginally increases by 4.27% when trade facilitation (ease of trade across borders) changes from low (1.9) to high (81.9). However, revenue decreases by 27.63% in contexts with medium competition and 44.37% in contexts with high competition when trade facilitation changes from low to high. These statistics confirm the acceptance of H2.

Furthermore, despite trade facilitation’s negative impact on SMEs’ performance, the panel regression estimator results and the ATET results of all PSM methods, confirm the positive impact of enabling tax administration and business licencing and registration regulations on SMEs’ performance (see Tables 5, 6, 7, 8, and 9). These results thus lead to the acceptance of this study’s third hypothesis (H3).

**Table 9:** ATET results of regression adjustment^a^

	Objective regulatory BE			Subjective regulatory BE		
	Ease of trade across borders	Ease of paying taxes	Ease of starting a business	Customs and trade regulations	Tax administration	Business licensing and permits
ATET	− 0.068	1.315***	1.739***	− 0.434***	0.225***	0.231***
	(0.061)	(0.048)	(0.051)	(0.069)	(0.051)	(0.062)
POM^b^ (enabling reg BE)	12.210***	11.591***	10.870***	12.180***	11.788***	11.792***
	(0.050)	(0.025)	(0.043)	(0.067)	(0.045)	(0.058)
Observations	18,685	19,454	21,170	17,728	16,586	16,946

Standard errors in parentheses

^***^p < 0.01

^**^p < 0.05

^*^p < 0.1

ATET is average treatment effect on the treated

^a^Outcome model is Poisson

^b^Potential outcome mean

The following covariates are included in all models: retained earnings, bank finance, access to finance, size of firm, age of firm, status of firm, human capital of O/M, corruption, informal firms, GDP per capita of country, and year of survey

### Discussion

This study’s central finding is that whilst enabling tax administration and business licensing and registration regulations improve African SMEs’ performance, trade facilitation decreases SMEs’ performance. Moreover, the institutional context of competition worsens trade facilitation’s negative impact on African SMEs’ performance.

These interesting findings present new insights into Africa’s institutional setting and BE.^8^ Trade facilitation’s usefulness for developing countries has been questioned with mixed findings (Siddiqui, 2015). On the one hand, Obuobi et al. (2022) found that trade facilitation increased FDI flows to African countries; on the other hand, Yameogo and Omojolaibi (2021) noted that trade facilitation in sub-Saharan Africa was detrimental to economic growth in the short term, even though it reduced poverty in the long term. Even so, the literature has not fully explored trade facilitation’s impact on particularly African SMEs’ performance. Our findings therefore fill an important gap in the literature, demonstrating trade facilitation’s detrimental impact on African SMEs’ performance.

In this regard, our findings seem plausible since SMEs are unique firms with challenges that are exacerbated in the African context. For instance, poor access to finance (Fowowe, 2017), corruption (d'Agostino et al., 2016), and weak institutions (Alhassan & Kilishi, 2019) impact African SMEs’ operations immensely. SMEs also find penetrating markets and competing against large firms challenging (Sitharam & Hoque, 2016), thus increased competition that may come with trade facilitation would be severely detrimental to African SMEs. Mutalemwa (2015) found that globalisation damaged African SMEs’ growth prospects. Fatoki (2014) argued that competition and high distribution costs led to the failure of new SMEs in South Africa. Moreover, though not focused on SMEs, Hunt et al. (2007) found that increased competition led to increased unemployment and hardships for firms operating in the clothing sectors of Algeria, Morocco, and Tunisia. It seems, therefore, that the benefits of trade facilitation and increased trade across borders do not reach SMEs in the African context. Increased competition from foreign firms (owing to trade facilitation) overrides any benefits to SMEs. Unlike large firms, SMEs do not have the capacity or leverage to compete against foreign firms; trade facilitation thus impedes their performance.

Quite interestingly, using corresponding objective (country-level) and subjective (firm-level) proxies of regulations, our findings confirm the hypotheses of this study, that is, the negative impact of trade facilitation as opposed to the positive impact of tax administration and business licencing regulations on African SMEs’ performance (Adeniyi & Imade, 2018; Devas & Kelly, 2001; Kamasa et al., 2020). Firm-level measures are often pitched against country-level measures because the latter cloud the heterogeneity in each country or even in regions within a country (Dethier et al., 2011; Dollar et al., 2005). Country-level measures are also presumed to not fully capture how different institutional deficiencies affect each unique firm (Straub, 2008). Nevertheless, our findings confirm that these supposedly opposing measures are complementary, at least as far as measuring BE regulations are concerned. In fact, the World Bank’s Doing Business measures are sometimes criticised for their weakness in correctly measuring regulations’ impacts in countries (McCormack, 2018). In any case, our empirical strategy of using the Doing Business measures (as objective measures of regulations) and subjective measures is interesting and contributes to the empirical literature, which other scholars may find useful.

### Robustness checks

An alternative measure of performance, profit, was used for the econometric analyses to confirm the results’ robustness. All results obtained from these robustness checks were statistically the same as those in the main analyses (see Appendix Tables 11, 12, 13, and 14).

## Conclusion and implications

This study aimed to extend the research on institutional context and BE regulations’ impact on African SMEs’ performance. Whilst enabling tax administration and business licensing and registration regulations improve African firms’ operations and performance (Adeniyi & Imade, 2018; Devas & Kelly, 2001; Kamasa et al., 2020), trade facilitation’s impact raises queries (Hunt et al., 2007; Siddiqui, 2015; Terzİ, 2010). Moreover, how trade facilitation impacts, particularly, African SMEs’ performance is missing in the literature to the best of our knowledge. Whether the institutional context of competition (from foreign firms) negatively moderates trade facilitation’s impact on African SMEs’ performance is also missing from the literature and thus represents an additional value of this contribution.

Using regressions and PSM methods on the latest cross-country African panel datasets from the WBES, our findings interestingly indicate that, whilst enabling tax administration and business licensing regulations improve SMEs’ performance, trade facilitation impedes African SMEs’ performance. Furthermore, we note that the institutional context of competition exacerbates trade facilitation’s negative impact on African SMEs’ performance.

These findings are interesting since they show that not all enabling regulations promoted in policies directed at SMEs in Africa and perhaps other developing countries benefit SMEs. SMEs have unique challenges and are unable to compete with foreign firms when trade across borders is liberalised in African countries. In such instances, trade facilitation counteracts its intended purpose of improving SMEs’ performance (Hunt et al., 2007; Siddiqui, 2015; Terzİ, 2010). Although trade facilitation improves the macroeconomy in African countries—such as increasing FDI inflows (Obuobi et al., 2022), improving some firms’ productivity (Mabugu & Mabugu, 2014; Teweldemedhin & van Schalkwyk, 2010), and reducing poverty (Yameogo & Omojolaibi, 2021)—there is substantial evidence that trade facilitation is detrimental to African SMEs’ performance.

This study makes three important contributions to the literature on institutional heterogeneity in Africa: First, it provides first-time simultaneous evidence of the varied impact of enabling BE regulations on African SMEs’ performance. To our knowledge, no study has examined such evidence regarding Africa. Second, this study offers new evidence demonstrating trade facilitation’s detrimental impact on African SMEs’ performance, which should be insightful to policymakers. Third, this study provides evidence that the institutional context of competition worsens trade facilitation’s detrimental impact on SMEs’ performance. Additionally, this study provides new evidence on the complementarity of objective country-level and subjective firm-level measures of regulations.

This study’s findings should interest policymakers, governments, and scholars, especially in Africa, since they present certain implications. First, these findings call for a recalibration of some regulatory policies. We suggest that initiatives to improve the regulatory framework in African countries be fine-tuned to benefit SMEs. For example, whilst the provision of an adequate business licensing and registration system greatly increases formality and improves firms’ performance (Alfaro & Chari, 2014; Demenet et al., 2016; Fernandes et al., 2018), trade facilitation must be carefully thought through and implemented in such a way that SMEs are not disadvantaged. This does not entail a full systematic approach to SME policy promotion as in the well-defined case of the European Smart Specialisation Strategy (McCann and Ortega-Argiles, 2016) but demonstrates the need to tailor regulatory interventions to gain the desired impact. Second, an implication which scholars should find useful is that country-level proxies of regulations consistently complement firm-level measures of regulations. Despite the supposed weakness of country-level measures (in this paper, the World Bank’s Doing Business measures), we note their consistent complementarity to firm-level measures.

Our study was limited by the sample of African countries used; conducting a similar study focused on other developing countries from other regions or emerging economies would therefore be insightful. It would also be insightful to conduct a study that compares the impact of BE regulations in developing countries with those in developed countries.

## Data Availability

The data used in this study is available at https://www.enterprisesurveys.org/en/survey-datasets.

## Appendix

Table 10.

Table 11.

Table 12.

Table 13.

Table 14.

**Table 10:** Regulation variables

Regulation	Objective (country-level) variables	Subjective (firm-level) variables
Trade facilitation	The DB ease-of-trading-across-borders score	The firm’s perception of the impact of customs and trade regulations on its operations
Tax administration	The DB ease-of-ease-of-paying-taxes score	The firm’s perception of the impact of tax administration on its operations
Business licensing and registration	The DB ease-of-starting-a-business score	The firm’s perception of the impact of business licencing and permit regulations on its operations

*DB*, World Bank’s Doing Business project

**Table 11:** Robustness regression results

Profit	Model 1	Model 2	Model 3
Ease of trading across borders		− 0.048***	0.033***
		(0.002)	(0.006)
Institutional context of competition (from foreign firms) × ease of trading across borders			− 0.001***
			(0.000)
Ease of paying taxes		0.012***	0.019***
		(0.002)	(0.002)
Ease of starting a business		0.083***	0.077***
		(0.002)	(0.002)
Customs and trade regulations		− 0.153***	− 0.172***
		(0.017)	(0.017)
Tax administration		0.059***	0.065***
		(0.017)	(0.017)
Business licensing and permits		0.116***	0.100***
		(0.017)	(0.017)
Retained earnings and internally gen. funds	0.042**	0.064***	0.068***
	(0.019)	(0.020)	(0.020)
Banks (public and private)	0.411***	0.316***	0.298***
	(0.040)	(0.045)	(0.044)
Access to finance	− 0.119***	− 0.078***	− 0.087***
	(0.013)	(0.014)	(0.014)
Size of firm	1.188***	1.073***	1.066***
	(0.019)	(0.019)	(0.019)
Age of firm	− 0.141***	− 0.176***	− 0.156***
	(0.027)	(0.028)	(0.028)
Status of firm	− 0.203***	− 0.262***	− 0.229***
	(0.020)	(0.021)	(0.021)
Human capital of O/M	0.148***	0.234***	0.222***
	(0.028)	(0.029)	(0.029)
Corruption	− 0.119***	− 0.121***	− 0.120***
	(0.013)	(0.014)	(0.014)
Informal firms	0.006	0.061***	0.057***
	(0.014)	(0.015)	(0.015)
Country	− 0.034***	− 0.074***	− 0.098***
	(0.003)	(0.003)	(0.004)
GDP per capita	0.246***	− 0.247***	− 0.325***
	(0.027)	(0.032)	(0.032)
Institutional context of competition (from foreign firms)	0.006***	− 0.020***	0.042***
	(0.001)	(0.001)	(0.004)
Constant	6.968***	9.416***	6.630***
	(0.249)	(0.267)	(0.321)
Wald Chi-square test	5868.57***	9173.87***	9468.82***
Observations	22,394	18,272	18,272
Number of firms	20,308	17,237	17,237

Standard errors in parentheses

^***^*p* < 0.01

^**^*p* < 0.05

^*^*p* < 0.1

model is random effects

**Table 12:** Robustness results—profit ATET results of nearest neighbour matching

	Objective regulatory BE			Subjective regulatory BE		
	Ease of trade across borders	Ease of paying taxes	Ease of starting a business	Customs & trade regulations	Tax administration	Business licensing and permits
Nearest neighbour (3)	− 1.855***	0.948***	1.714***	− 0.307***	0.156***	0.209***
	(0.093)	(0.058)	(0.055)	(0.072)	(0.055)	(0.073)
Observations						
Total raw	17,620	18,331	19,845	16,626	15,525	15,810
Total matched	10,942	13,262	17,556	27,324	20,694	25,124
Treated matched	5471	6631	8778	13,662	10,347	12,562
Control matched	5471	6631	8778	13,662	10,347	12,562

standard errors in parentheses

^***^*p* < 0.01

^**^*p* < 0.05

^*^*p* < 0.1

ATET is average treatment effect on the treated

The following covariates are included in all models: retained earnings, bank finance, access to finance, size of firm, age of firm, status of firm, human capital of O/M, corruption, informal firms, GDP per capita of country, and year of survey

**Table 13:** Robustness results—profit ATET results of inverse probability weighting

	Objective regulatory BE			Subjective regulatory BE		
	Ease of trade across borders	Ease of paying taxes	Ease of starting a business	Customs and trade regulations	Tax administration	Business licensing and permits
ATET	− 0.781***	0.909***	1.367***	− 0.311***	0.139**	0.290***
	(0.070)	(0.053)	(0.106)	(0.089)	(0.060)	(0.076)
POM^a^ (Enabling reg BE)	12.112***	11.193***	10.749***	11.433***	11.219***	11.109***
	(0.056)	(0.029)	(0.102)	(0.087)	(0.054)	(0.073)
Observations						
Total raw	17,620	18,331	19,845	16,626	15,525	15,810
Total weighted	17,620	18,331	19,845	16,626	15,525	15,810
Treated weighted	9168.7	9142.3	9444.0	8026.3	7690.6	7946.8
Control weighted	8451.3	9188.7	10,401.0	8599.7	7834.4	7863.2

standard errors in parentheses

^***^*p* < 0.01

^**^*p* < 0.05

^*^*p* < 0.1

ATET is average treatment effect on the treated

The following covariates are included in all models: retained earnings, bank finance, access to finance, size of firm, age of firm, status of firm, human capital of O/M, corruption, informal firms, GDP per capita of country, and year of survey

**Table 14:** Robustness results—profit ATET results of regression adjustment^a^

	Objective regulatory BE			Subjective regulatory BE		
	Ease of trade across borders	Ease of paying taxes	Ease of starting a business	Customs and trade regulations	Tax administration	Business licensing and permits
ATET	− 0.602***	0.981***	1.651***	− 0.419***	0.095*	0.201***
	(0.065)	(0.051)	(0.056)	(0.073)	(0.053)	(0.067)
POM^b^ (enabling reg BE)	11.933***	11.121***	10.465***	11.541***	11.263***	11.197***
	(0.052)	(0.026)	(0.048)	(0.070)	(0.048)	(0.064)
Observations	17,620	18,331	19,845	16,626	15,525	15,810

standard errors in parentheses

^***^*p* < 0.01

^**^*p* < 0.05

^*^*p* < 0.1

ATET is average treatment effect on the treated

^a^Outcome model is Poisson

^b^Potential outcome mean

The following covariates are included in all models: retained earnings, bank finance, access to finance, size of firm, age of firm, status of firm, human capital of O/M, corruption, informal firms, GDP per capita of country, and year of survey

## Footnotes

1 Business licensing and registration regulation includes easy access to affordable business registration services, among others.

2 In a broader sense, Siddiqui (2015) questioned the usefulness of universal trade facilitation, which international institutions strongly advocate for developing countries. Calling for a rethink, he argued that trade facilitation shrinks development, self-determination, and economic sovereignty in developing countries.

3 For instance, African SMEs have limited prospects of exporting their products in large batches and may find arranging collective responses to large international orders difficult. Other African SMEs may find meeting international standards and certifications difficult.

4 Khanna and Palepu (1997) explained that institutional voids are the absence or underdevelopment of institutions that support markets. They are macroeconomic situations where arrangements that support markets are weak or do not perform as expected (Aidis, 2005; Mair & Marti, 2009; Puffer, McCarthy, & Boisot, 2010; Stal & Cuervo-Cazurra, 2011). Institutional theory dates back to Selznick (1957) and North (1990), who suggested that firms are adaptive structures shaped in reaction to their external environment and the actions of participants within the firm.

5 Ethiopia was excluded because its data set was missing a panel ID variable.

6 The WBES classifies firms in Africa as micro firms (1–4 employees), small firms (5–19 employees), medium firms (20–99 employees), and large firms (100 + employees).

7 Globalisation involves the creation of networks of connections among actors across geographical boundaries, which promotes a flow of ideas, people, capital, goods, and information (Dreher, 2006).

8 The positive impact of enabling tax administration and business licencing regulations on SMEs’ performance in this study confirms the well-established claims in the literature (Devas & Kelly, 2001; Kamasa et al., 2020).
